# Impact of Heat-Killed *Lactobacillus casei* Strain IMAU60214 on the Immune Function of Macrophages in Malnourished Children

**DOI:** 10.3390/nu12082303

**Published:** 2020-07-31

**Authors:** Luz María Rocha-Ramírez, Beatriz Hernández-Ochoa, Saúl Gómez-Manzo, Jaime Marcial-Quino, Noemí Cárdenas-Rodríguez, Sara Centeno-Leija, Mariano García-Garibay

**Affiliations:** 1 Unidad de Investigación en Enfermedades Infecciosas, Hospital Infantil de México Federico Gómez, Secretaría de Salud Dr. Márquez No. 162, Col Doctores, Delegación Cuauhtémoc, Ciudad de México 06720, Mexico; 2 Laboratorio de Investigación en Inmunoquímica, Hospital Infantil de México Federico Gómez, Secretaría de Salud. Dr. Márquez No. 162, Col Doctores, Delegación Cuauhtémoc, Ciudad de México 06720, Mexico; beatrizhb_16@hotmail.com; 3 Laboratorio de Bioquímica Genética, Instituto Nacional de Pediatría, Secretaria de Salud, Ciudad de México 04530, Mexico; saulmanzo@ciencias.unam.mx; 4 Consejo Nacional de Ciencia y Tecnología (CONACYT), Instituto Nacional de Pediatría, Secretaría de Salud, Ciudad de México 04530, Mexico; jmarcialq@ciencias.unam.mx; 5 Laboratorio de Neurociencias, Instituto Nacional de Pediatría, Secretaría de Salud, Ciudad de México 04530, Mexico; noemicr2001@yahoo.com.mx; 6 Consejo Nacional Ciencia y Tecnologia (CONACYT), Laboratorio de Agrobiotecnología, Tecnoparque CLQ, Universidad de Colima, Carretera Los Limones-Loma de Juárez, Colima 28629, Mexico; scenteno0@ucol.mx; 7 Departamento de Ciencias de la Alimentación, Unidad Lerma, Departamento de Biotecnología, Unidad Iztapalapa, Universidad Autónoma Metropolitana, Av. San Rafael Atlixco No. 186. Col Vicentina, Ciudad de México 09340, Mexico; jmgg@xanum.uam.mx

**Keywords:** malnourished, children, macrophage, immunity, cytokines, burst respiratory, phagocytosis, probiotics, *Lactobacillus*

## Abstract

Malnutrition is commonly associated with immunological deregulation, increasing the risk of infectious illness and death. The objective of this work was to determine the in vitro effects of heat-killed *Lactobacillus casei* IMAU60214 on monocyte-derived macrophages (MDMs) from well-nourished healthy children, well-nourished infected children and malnourished infected children, which was evaluated by an oxygen-dependent microbicidal mechanism assay of luminol-increase chemiluminescence and the secretion of tumor necrosis factor (TNF-α), interleukin (IL-1β), IL-6 and IL-10, as well as phagocytosis using zymosan and as its antibacterial activity against *Salmonella typhimurium*, *Escherichia coli* and *Staphylococcus aureus*. We found that reactive oxygen species (ROS), secretion cytokines (TNFα, IL-1β, IL-6 and IL-10 levels), phagocytosis and bactericidal capacity increased in all groups after pre-treatment with heat-killed *L. casei* IMAU60214 at a ratio of 500:1 (bacteria:MDM) over 24 h compared with MDM cells without pre-treatment. The results could indicate that heat-killed *L. casei* IMAU60214 is a potential candidate for regulating the immune function of macrophages.

## 1. Introduction

Malnutrition in children is a worldwide health problem; furthermore, the presence of bacterial and viral infections is a common complication of this condition [1]. Approximately 25% of children younger than 5 years of age show a malnourished state in developing countries [2]. Malnutrition remains an important risk factor for predisposition to respiratory and intestinal infections [3,4,5,6]. Protein energy malnutrition (PEM) is also an immunodeficiency that is strongly associated with susceptibility to infections [7,8]. It has been proposed that there is a cause–consequence relationship between immune alterations and malnutrition; however, the effect of cellular alterations on the immune system in malnourished patients has not yet been sufficiently studied [9,10]. In malnourished patients, alterations in the innate immune system have been observed in the epithelial barrier of the skin and intestinal system along with poor granulocyte bactericidal activity, less circulating dendritic cells and reduced complement proteins [11,12]. In addition, in a PEM murine model, leucopenia and a severe reduction of cell numbers in organs such as the spleen and bone marrow before and after stimulus with lipopolysaccharide (LPS) have been observed [13]. Furthermore, Fock et al. [13] demonstrated that interleukin-10 (IL-10) levels increased in malnourished animals inoculated with LPS, suggesting a poor immune response to LPS, because IL-10 has an antagonistic effect on pro-inflammatory cytokines. It has also been observed that macrophage function is significantly affected, limiting their ability to eliminate intracellular pathogens [14]. Moreover, studies in malnourished children have shown low levels of CD69+, CD4+ and CD8+ expression in lymphocytes and an increased level of cytokines associated with Th 2 response (IL-4 and IL-10 levels) and decreased cytokine profiles related to the Th 1 response (interferon IFN-ү, IL-2 levels) in lymphocytes from this population. The results obtained suggest that a state of malnourishment alters the ability of CD8+ and CD4+ cells to produce IL-2, IFN-γ, IL-4 and IL-10 as a response to stimulus [15,16]. Besides this, in aged hosts, there is a decrease in primary antibody response activity, which leads to the production of antibodies with lower antigenic affinity [17].

It has been recognized that in immunocompetent hosts, the response of macrophages is initiated after pathogen recognition through toll-like receptors (TLRs) and mostly consists of phagocytosis, pro and anti-inflammatory cytokine release, oxidative burst activation and regulation by the differentiation of macrophages M1 and/or M2 [18]. In contrast, in immunocompromised hosts, such as in the case of malnourished patients, it is well documented that there are failures in the functionality of macrophages [19].

In experimental animal models [20] and in human clinical trials [21,22], positive effects of heat-killed bacteria in different conditions have been observed, such as probiotic administration in neonates, without the risks associated with live bacteria [23,24]. Preparations with dead or live microorganisms and metabolites have shown good biological properties in terms of reestablishing intestinal homeostasis; in many cases, these effects are similar to those obtained with the administration of live bacteria [20,25]. On the other hand, bacterial inactivation by heat treatment (heat-killed bacteria) can liberate bacterial components with immunomodulatory effects by way of many biological mechanisms such as an increase in IgA salivary production [25] and the modulation of host T-cell responses [26], which also have been shown to modulate the inflammatory response by regulating IL-10, human β-defensin and other pro-inflammatory cytokines [27].

In relation to preparations with heat-killed bacteria, it has been proposed that lipoteichoic acids, peptidoglycans or exopolysaccharide (EPS) bacterial components are responsible for the beneficial properties of the preparations [20,28,29]. To date, the effects of some heat-killed lactobacilli on innate immunity cells—particularly macrophages—have not yet been studied in malnourished children and thus represent a target of interest for research.

The use of immunomodulators such as heat-killed lactobacilli could help to enhance the immune response, which represents a target of interest for immunocompromised hosts, including malnourished children, where it is necessary to minimize undesirable effects. In this sense, in a study carried out in adults (50–70 years of age), it was observed that the use of heat-killed *Lactobacillus gasseri* could enhance immune system activity through an increase in CD8+ lymphocyte levels [30]. In addition, in vitro studies in murine models have shown that a cell-free extract of lactic acid bacteria (LAB) increases phagocytic activity by stimulating the expression of some cytokines as well as antibodies of the IgG isotype [31]. A great variability exists in the ability of heat-killed LAB strains to enhance the function of the immune response, and this type of response has been considered to be species-specific. In vitro studies on mouse macrophage cells using a heat-killed combination of LAB (*Lactobacillus acidophilus*, *Lactobacillus plantarum*, *Lactobacillus fermentum* and *Entercoccus faecium*) showed an increase in the production of IL-12, while the cell-wall preparations decreased the invasion of *Salmonella* on Caco-2 cell line and mouse macrophage cells [32]. Another report demonstrated that the heat-killed *Lactobacillus casei* strain Shirota (LcS) was able to increase IL-1β and induced antimicrobial peptide human β-defensine-2 (hBD-2) in Caco-2 colon intestinal epithelial cells, and LcS partially stimulated tumor necrosis factor alpha (TNF-α) [33].

Finally, a recent study on RAW264.7 murine macrophage cells reported that treatment with heat-killed *Lactobacillus plantarum* KCTC 133114BP increased the production of TNF-α and IL-6, indicating that LBP can be proposed to be an immune-enhanced functional food agent [34].

Despite the studies conducted in this field, none of these have been translated into human malnutrition, and it is not clear what effect probiotic treatment has on the recovery of macrophage function, especially under stress conditions such as infection and malnutrition.

In this work, we determine the effect of heat-killed *L. casei* IMAU60214 on the function of macrophages derived from monocytes of well-nourished healthy (WNH), well-nourished infected (WNI) and malnourished infected (MNI) children, evaluating innate functions through reactive oxygen species (ROS) secretion, the release of pro- and anti-inflammatory cytokines (TNF-α, IL-β, IL-6 and IL-10 levels), phagocytosis and bactericidal capacity against an intracellular pathogen such as *Salmonella typhimurium* and extracellular pathogens such as *Escherichia coli* and *Staphylococcus aureus*. We found that the production of ROS, secretion cytokines, phagocytosis and bactericidal capacity increased in all groups after pre-treatment with heat-killed *L. casei* IMAU60214 with respect to MDM cells without pre-treatment. These results could indicate that heat-killed *L. casei* IMAU60214 is a potential candidate for regulating the immune function of macrophages. Finally, it is interesting to mention that this is the first time that the effect of heat-killed *L. casei* IMAU60214 on the immune function of macrophages in malnourished infected children has been characterized.

## 2. Materials and Methods

### 2.1. Study Subjects and Biological Sample Collection

This study was evaluated and approved by the Institutional Ethics and Research Committees of the Children’s Hospital, Mexico (protocol number HIM/2014/013 SSA). After obtaining informed consent from parents of each participating child, peripheral blood was collected within 24 h of hospital admission in a Vacutainer test tubes containing ethylenediamine–tetraacetic acid (EDTA) (Becton-Dickinson, Le Pont de Claix, France). As inclusion criteria, in this experimental study, we included children of both sexes aged between 6 months and 5 years old, classified into three groups: well-nourished healthy children (WNH), well-nourished infected children (WNI) and malnourished-infected children (MNI) with a deficit in nutritional status of weight and height according at the age. As exclusion criteria, children with immunosuppressive drug treatments (steroids or cancer treatment), patients living with Human Immunodeficiency Virus (HIV), cancer or other immunodeficiencies were excluded. Blood samples of 45 children of both sexes were included. The relationships of weight and height in WNH and WNI children groups were normal according to age, while the severity of acquired malnutrition of MNI children was assessed by clinical signs and weight/height relationship deficit according to the criteria for Mexican children and some criteria of the malnutrition protocol of the World Health Organization (WHO) [35,36]. WNI and MNI children groups were identified with acute bacterial infection, corroborated by clinical symptoms as fever, hypothermia, the identification of the infectious site, abdominal pain and laboratory tests such as hematic biometry, leukocytosis, leukopenia, lymphocytosis, neutrophilia and bacterial cultures; most of them were hospitalized for acute gastrointestinal and respiratory bacterial infections and other infections (Table 1)**.**

### 2.2. Culture of Monocyte-Derived Macrophage (MDM)

The blood samples of the groups of children included in this study (WNH, WNI and MNI) were diluted to a 1:1 volume with physiological saline solution, and mononuclear cells were separated using gradient centrifugation [37]. Briefly, the interface containing mononuclear cells was washed twice with physiological saline solution; immediately afterwards, the cells were counted, and cell viability was evaluated staining with trypan blue (Gibco BRL, Rockville, MD, USA). The mononuclear cell packet was adjusted to a final concentration of 10 × 10^6^ mononuclear cells/mL in Roswell Park Memorial Institute (RPMI) 1640 medium (Gibco BRL, Rockville, MD, USA) plus 25% human serum pool (PSH). Subsequently, 40 µL of this cell suspension was placed on coverslips of 0.9 mm in diameter and incubated for 2 h in a 5% CO_2_ atmosphere. Then, cells were washed four times with warm Hank´s balanced salt solution (HBBS; Gibco BRL, Rockville, MD, USA) to remove non-adherent cells. Adherent monocytes cells were maintained in cultures for 7 days for differentiation to macrophages; the purity of the population under these culture conditions was greater than 90%, according to the morphology of the cells.

### 2.3. Bacterial Strain

*L. casei* IMAU60214 is a fermented milk isolate previously characterized by Cruz-Guerrero [38]. The bacteria were grown in Man–Rogosa–Sharpe (MRS) broth (BD, Difco Laboratories, Franklin Lakes, NJ, USA) overnight at 37 °C under anaerobic conditions, harvested by centrifugation and washed three times in physiological saline solution. The number of colony forming units (CFU)/mL was determined by cell counting in a dilution of base 10, and the concentration was adjusted to 1 × 10^9^ CFU/mL. Heat-killed bacterial cells of *L. casei* IMAU60214 were prepared by heating at 85 °C for 10 min. *Staphylococcus aureus* American Type Culture Collection (ATCC) 29213 (KIWK-STIK^TM^, MicroBioLogics, St. Cloud, MN, USA), *Escherichia coli* ATCC 35,218 (KIWK-STIK^TM^, MicroBioLogics, St. Cloud, MN, USA) and *Salmonella typhimurium* ATCC 14,028 (KIWK-STIK^TM^, MicroBioLogics, St. Cloud, MN, USA) strains were grown overnight in tryptic soy broth (BD, Difco Laboratories) at 37 °C. Thereafter, the cells were centrifuged to 5000× *g* for 5 min, washed in sterile physiological saline solution and normalized to a density of ~10^8^ CFU/mL.

### 2.4. Pre-Treatment of MDM Cells with Heat-Killed L. casei IMAU60214

MDM cells from the three groups included in this study (WNH, WNI and MNI) were treated according to the method reported by Rocha-Ramirez et al. [39]. Briefly, MDM cells were pre-incubated with heat-killed *L. casei* IMAU60214 at a ratio of 500:1 (Bacteria: MDM) for 24 h in a 5% CO_2_ atmosphere. The cells were then washed four times with warm Hanks solution. Subsequently, supplemented RPMI-1640 medium 10% Fetal Bovine Serum (FBS) (Gibco BRL, Rockville, MD, USA) was added to the MDM cells. Afterwards, the cells were then incubated at 37 °C for 18 h in a 5% CO_2_ atmosphere, before the addition of the zymosan (Sigma-Aldrich, St. Louis, MO, USA) stimulus. Non-treated MDM cells with heat-killed *L. casei* IMAU60214 were incubated for 24 h in a 5% CO_2_ atmosphere. Afterwards, these MDM cells were washed under the same conditions as the treated MDM cells and were used as negative controls.

### 2.5. Chemiluminescence

The MDM cells for the ROS secretion assay were treated in the same way as in Section 2.4 (Materials and Methods); the ROS secretion was determined by a chemiluminescence assay, with amplified luminol (Sigma-Aldrich, St. Louis, MO, USA) detected at 37 °C in an LKB-1264 luminometer. Briefly, the coverslips with monolayers of MDM cells were individually placed with the aid of tweezers in polypropylene tubes and immediately covered with 1 mL of reaction solution containing warm Hanks solution, luminol (0.8 × 10^−4^ M) and horseradish peroxidase (Sigma-Aldrich, St. Louis, MO, USA). Opsonized zymosan was used as a stimulus for ROS secretion and prepared by the addition of 10% of serum human AB and incubated for 30 min at 37 °C, washed and centrifuged at 5000× *g* for 5 min and finally suspended in Hanks solution. The assays were performed in duplicate, with and without the pre-treatment of heat-killed *L. casei* IMAU60214.

### 2.6. Cytokine Secretion

Cytokine levels of TNF-α, IL-1β, IL-6 and IL-10 in the culture supernatants from treated and un-treated MDM cells (from three groups: WNH, WNI and MNI) were measured with enzyme-linked immunosorbent assay (ELISA) technique kits following the manufacturer´s guidelines (BD Biosciences, kit Opt EIA PharMingen, San Diego, CA, USA). Absorbance at 450 nm was read on a microplate reader (Fluoroskan Ascent RThermo-Labsystems), and cytokine levels were calculated by extrapolation to recombinant cytokine curves performed in duplicate samples.

### 2.7. Examination of the Phagocyte Function of MDM Cells

For phagocytosis assay, a stock zymosan was prepared at a concentration of 1 mg/mL in saline solution, and different dilutions were created to adjust the concentration to 1 × 10^6^ zymosan, which was counted in a Neubauer chamber. A zymosan suspension was then opsonized with 10% AB human serum in Hanks solution and incubated at 37 °C for 30 min in a 5% CO_2_ atmosphere. Subsequently, it was washed twice by centrifugation at 5000× *g* for 5 min. Afterwards, the zymosan was added to the MDM cells of the different study groups (WNH, WNI and MNI) at a ratio of 10:1 (zymosan:MDM) and incubated with Hanks solution for 1 h in a 5% CO_2_ atmosphere. Phagocytosis was terminated by vigorously washing off non-ingested zymosan with warm Hanks solution two times. Glass coverslips were air-dried for 10 min and stained using a hematological staining kit (Hycel, Mexico). Later, the coverslips were washed with water and air-dried and mounted on glass slides using Entellan (Merck, Mexico). The percentage of MDM ingesting zymosan and the mean amount of ingested zymosan per phagocytic cell were evaluated under light microscopy at 1000x magnification using a Nikon photomicroscope (Nikon Canada Inc., Richmond, BC, Canada) by counting the amount of zymosan per cell for at least 50 MDM cells by sample. The percentage of phagocytosis activity was calculated according to the following formula: percentage phagocytosis activity = number of MDM cells with zymosan internalized per 50 MDM cells. The assays were performed in duplicate, with and without the pre-treatment of heat-killed *L. casei* IMAU60214.

### 2.8. Activity Bactericidal Assay

The gentamicin protection assay on MDM cells was performed for *Staphylococcus aureus* (ATCC 29213), *Escherichia coli* (ATCC 35218) and *Salmonella typhimurium* (ATCC 14028) strains at a multiplicity of infection (MOI) of 10:1 (Bacteria:MDM) and incubated at 37 °C for 120 min. After this incubation, the cells were washed gently three times with warm Hanks solution; to eliminate extracellular bacteria, gentamicin at 200 µg/mL was added, and the MDM cells were incubated at 37 °C for 1 h. Afterwards, the medium was replaced with gentamicin at 20 µg/mL and the MDM cells were incubated again for 1 h to ensure the complete elimination of extracellular bacteria. Afterwards, the MDM monolayers were washed three times with warm Hanks solution and the cells were lysed with 100 μL solution of 1% triton X-100 and incubated for 10 min. Finally, the MDM lysates were diluted 10-fold, plated on Luria–Bertani (LB, Bioxon, Mexico State, Mexico) agar and incubated overnight at 37 °C. The number of viable intracellular bacteria was determined by the standard colony counting technique. The assay was performed in duplicate.

### 2.9. Statistical Analysis

To compare the change which occurred after pre-treatment with heat-killed *L. casei* IMAU60214 on MDM cells, the paired Student´s *t*-test was used within groups. Data are shown as the mean and standard deviation of two independent experiments. The criterion for statistical significance was defined as at *p* < 0.05.

## 3. Results

### 3.1. Effect of Heat-Killed L. casei IMAU60214 on the Secretion of ROS on MDM Cells from WNH, WNI and MNI Chlidren

**Figure 1:**
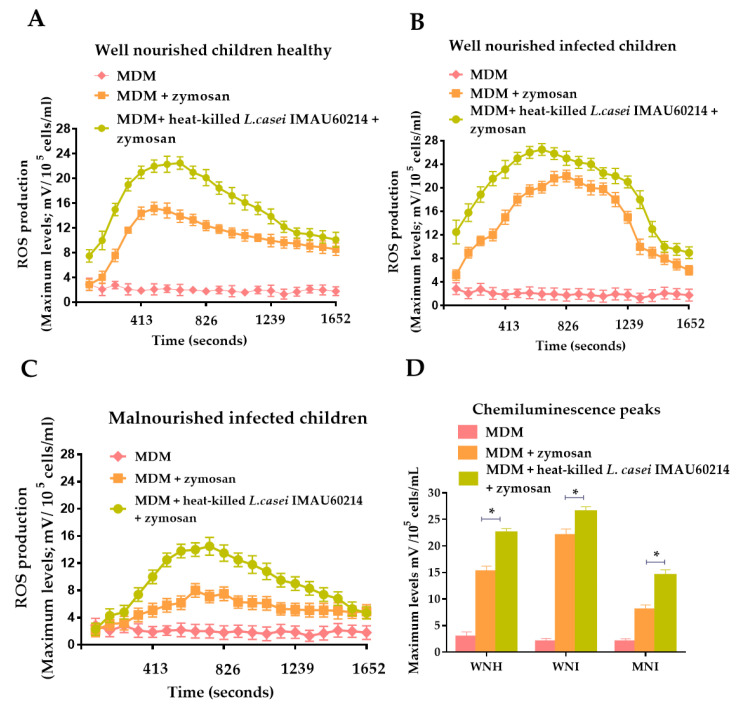
Effect of heat-killed *L. casei* IMAU60214 on reactive oxygen species (ROS) secretion by monocyte-derived macrophage (MDM) cells from well-nourished healthy (WNH), well-nourished infected (WNI) and malnourished infected (MNI) children. MDM cells 5 × 10^5^/mL in Roswell Park Memorial Institute (RPMI)-1640/10% FBS were pre-treated with heat-killed *L. casei* IMAU60214 (500:1, Bacteria: MDM) for 24 h. ROS secretion was measured with luminol-amplified chemiluminiscence assay: (**A**) kinetic curve of chemiluminescence for WNH children, (**B**) WNI children and (**C**) MNI children. (**D**) Maximum peak of chemiluminescence in ROS secretion at 1 h. Results are expressed as mean ± SD (*n* = 15) from duplicate tubes from MDM cells with and without pre-treatment of heat-killed *L. casei* IMAU60214; the bars with asterisks indicate significant differences between experimental conditions, determined by Student t paired test, * *p* < 0.05.

The immunostimulatory activity of heat-killed *L. casei* IMAU60214 through ROS secretion was investigated in vitro on MDM cells from the WNH, WNI and MNI children groups. As shown in Figure 1, the pre-treatment with heat-killed *L. casei* IMAU60214 increased the secretion of ROS in all groups of MDM cells to different extents compared with the basal secretion in the non-treated MDM cells. In addition, different peaks in ROS production kinetics (chemiluminescence peaks of 22.5, 26.5 and 14.5 mV for WNH, WNI and MNI children, respectively) were observed in the three study groups after pre-treatment with heat-killed *L. casei* IMAU60214 (Figure 1A–C). The maximum observed level of ROS production was reached between 660 s and 743 s (11 and 12 min) after adding the zymosan stimulus. With respect to the untreated MDM cells of MNI children with heat-killed *L. casei* IMAU60214, which were stimulated with zymosan, a maximum level of ROS production of 8 mV was observed; for the MDM cells pretreated with heat-killed *L. casei* and stimulated with zymosan, the determined ROS production was 14.5 mV, indicating an increase of 1.8-fold after the treatment with heat-killed *L. casei* IMAU60214 (Figure 1C). In contrast, the increase of ROS production in the other two groups was lower: The groups of WNH and WNI children exhibited an increase of 1.4-fold (from 15.1 mV to 22.5 mV) and 1.2-fold (from 22 mV to 26.5 mV), respectively (Figure 1D). These results indicate that pre-treatment with heat-killed *L. casei* IMAU60214 activates the ROS production in a greater proportion in the MDM cells of MNI children.

### 3.2. Heat-Killed L. casei IMAU60214 Induced Levels of Cytokine Secretion in Vitro on MDM Cells from WNH, WNI and MNI Chlidren

**Figure 2:**
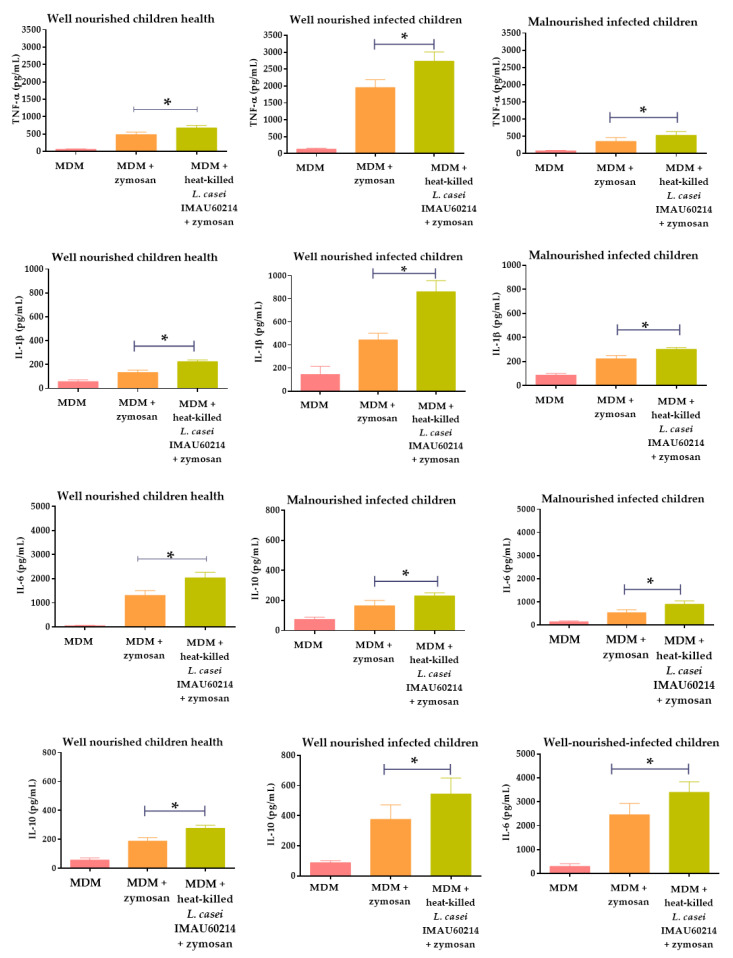
Effects of heat-killed *L. casei* IMAU60214 on cytokine production by MDM cells. The production of tumor necrosis factor (TNF-α), interleukin (IL)-1β, IL-6 and IL-10 were measured in the supernatant by immunosorbent assay (ELISA). All values are shown as mean ± SD (*n* = 15) from duplicate assays from MDM cells with and without pre-treatment of heat-killed *L. casei* IMAU60214. *p*-Values were calculated with the Student’s t paired test. * *p* < 0.05, Significant differences show the change which occurred after pre-treatment with heat-killed *L. casei* IMAU60214.

Cytokine-producing MDM cells pre-treated with heat-killed *L. casei* IMAU60214 at a relation of 500:1 (Bacteria: MDM) are shown in Figure 2. The cytokines levels of TNF-α, IL-1β, IL-6 and IL-10 were measured to represent pro and anti-inflammatory cytokines, respectively. The secretion of evaluated cytokines increased from 1.4 to 2-fold in all groups of MDM cells from WNH, WNI and MNI children groups after pre-treatment with heat-killed *L. casei* IMAU60214, with respect to the basal secretion in the absence of treatment. It is noted that the pre-treatment of MDM cells from MNI children with heat-killed *L. casei* IMAU60214 was not able to reach the highest levels of secretion shown for MDM cells from the WNI children group. However, we observed that the MDM cells of the MNI group show lower basal cytokine secretion rates for TNF-α (352 pg/mL), IL-β (222 pg/mL), IL-6 (535 pg/mL) and IL-10 (165 pg/mL), respectively, with respect to the WNI group. These results could indicate that, due to the malnutrition state of patients, the MDM cells do not respond adequately to the stimulus of zymosan for cytokine secretion. However, when the MDM cells from MNI children were pretreated with heat-killed *L. casei* IMAU60214, increases in the secretion of cytokines of 1.5, 1.4, 1.7 and 1.4-fold were achieved for TNF-α, IL-β, IL-6 and IL-10, respectively, as shown in Figure 2. This same increase in the secretion of cytokines was observed in the MDM cells from WNH and WNI children, where increases of about 1.5 to 2 times were obtained for the MDM cells pre-treated with heat-killed *L. casei* IMAU60214, even though basal cytokine secretion was higher with respect to the MNI children group.

### 3.3. Heat-Killed L. casei IMAU60214 Increase the Phagocytic Function In Vitro of MDM Cells from WNH, WNI and MNI Chlidren

**Figure 3:**
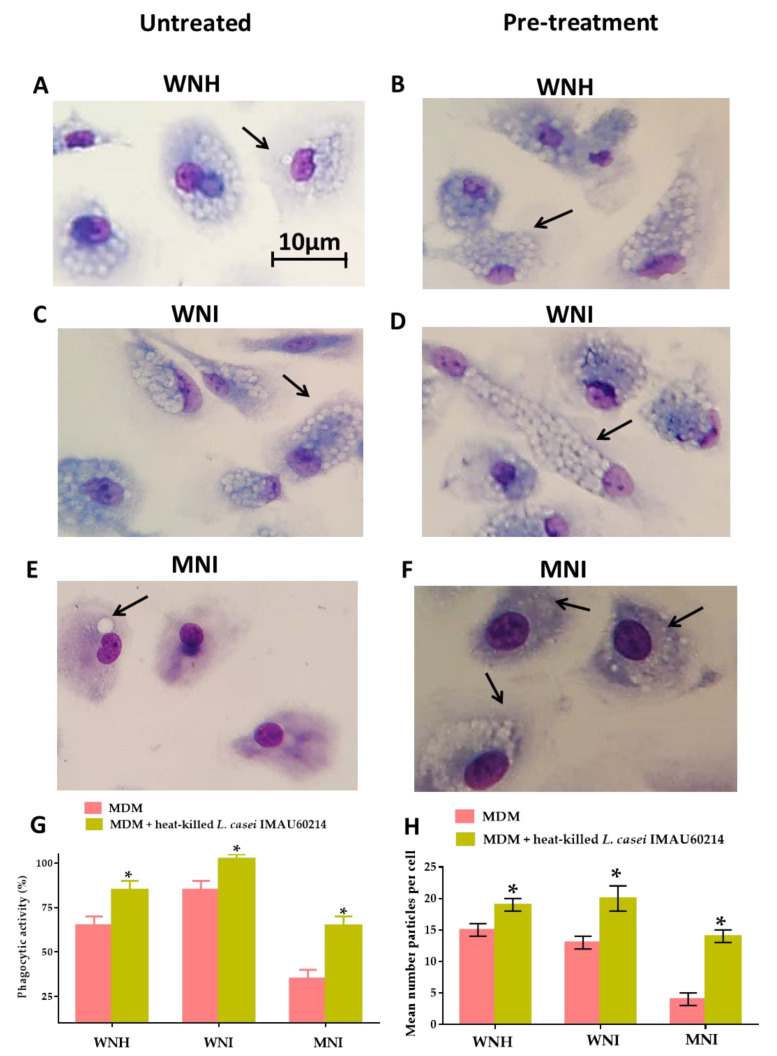
Heat-killed *L. casei* IMAU60214 increases the phagocytic function of MDM cells for WNH, WNI and MNI children. MDM cells were pretreated with heat-killed *L. casei* IMAU60214 at a ratio of 500:1 (Bacteria: MDM) for 24 h; zymosan was added to MDM cells at a ratio of 10:1. Representative photomicrographs show the phagocytosed zymosan by MDM cells. (**A**) WNH children untreated, (**B**) WNH children with pre-treatment, (**C**) WNI children untreated, (**D**) WNI children with pre-treatment, (**E**) MNI children untreated, (**F**) MNI children with pre-treatment. The arrow in panel (**A**,**C**,**E**) indicates an intracellular zymosan within the phagosome. The arrow in panel (**B**,**D**,**F**) shows the numerous vacuolations typically observed in activated MDM cells. (**G**) The percentage of phagocytic activity was calculated by the following formula: percentage phagocytic activity = number of MDM cells with zymosan internalized per 50 MDM cells, (**H**) mean amount of zymosan per cell. All values are shown as mean ± SD (*n* = 15) from duplicate assays from MDM cells with and without pre-treatment with heat-killed *L. casei* IMAU60214. * *p*-Values were calculated with Student’s t paired test. * *p* < 0.05, significant difference.

To determine the effect of heat-killed *L. casei* IMAU60214 on the MDM cells’ phagocytic activity, the MDM cells were pre-treated with heat-killed *L. casei* IMAU60214 for 24 h and challenged with zymosan for 1 h. In Figure 3, a representative photomicrograph of zymosan particles phagocytosed by untreated MDM cells from WNH, WNI and MNI children (Figure 3A,C,E) and pre-treatment with heat-killed *L. casei* IMAU60214 (Figure 3B,D,F) are shown; furthermore, the figure shows that the number of phagocytized zymosan was higher when MDM cells were pre-treated with heat-killed *L. casei* IMAU60214 in all groups (WNH, WNI and MNI). It is important to note that a malnutrition state in MNI children affects the phagocytic activity of MDM cells, since MDM from WNI children showed 85% phagocytic activity, while this was only 35% in MNI children (Figure 3G). When MDM cells were pre-treated with heat-killed *L. casei* IMAU60214, an increase in the percentage of phagocytic activity was observed in the three study groups, reaching 85%, 100% and 65% of phagocytic activity for WNH, WNI and MNI, respectively (Figure 3G). In addition, we observed that the mean number of zymosan phagocytosed per untreated MDM cell in the WNH and WNI children groups were 15 and 13 zymosan/cell, respectively, while that in the MNI children group was only 4 zymosan/cell (Figure 3H). When the MDM cells were pre-treated with heat-killed *L. casei* IMAU60214, an increase in the number of phagocytosed zymosan was observed, especially in the MNI group, where an increase of 3.5-fold (14 zymosan/cell) was reached, while in the WNH and MNI groups, an increase of 1.2 and 1.5-fold was observed, corresponding to 19 and 20 zymosan/cell, respectively (Figure 3H).

### 3.4. Heat-Killed L. casei IMAU60214 Increases the In Vitro Bactericidal Activity of MDM Cells from WNH, WNI and MNI Chlidren

**Figure 4:**
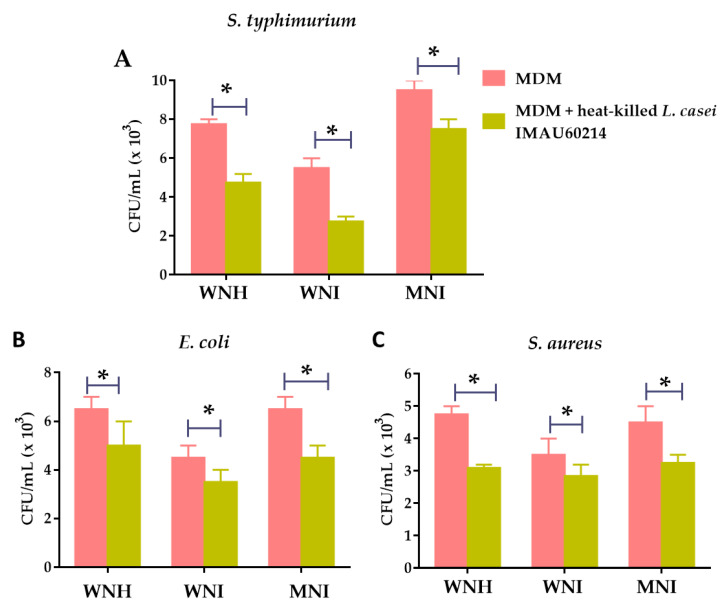
Effect of heat-killed *L. casei* IMAU60214 on the bactericidal activity of MDM cells from WNH, WNI and MNI children. MDM cells were pretreated or untreated with heat-killed *L. casei* IMAU60214 (500:1, Bacteria: MDM) for 24 h and then challenged with (**A**) *S*. *typhimiurium*, (**B**) *E. coli* and (**C**) *S. aureus* for 120 min; after that, they were washed and incubated with gentamicin to eliminate extracellular bacteria. Data are shown as CFU/mL recovered after MDM lysis. Each bar shows mean of triplicates ± SD of two independent experiments (*n* = 15). Asterisk indicates significant differences (* *p* < 0.05) between each condition analyzed with the treatments (Student’s t paired test).

The effect of pre-treatment with heat-killed *L. casei* IMAU60214 on the bactericidal activity of MDM cells from all experimental groups was determined. To this end, MDM cells from WNH, WNI and MNI children were challenged with *S. typhimurium*, *E. coli* and *S. aureus* strains, as described in the Materials and Methods section. As shown in Figure 4, when the MDM cells were pre-treated with heat-killed *L. casei* IMAU60214, a decrease in recovered CFU was observed for the pathogens *S. typhimurium* (Figure 4A), *E. coli* (Figure 4B) and *S. aureus* (Figure 4C) strains, with respect to the untreated MDM cells in the WNH, WNI and MNI children groups. These results reveal that the MDM cells of the three groups increased their intracellular bactericidal capacity after being treated with heat-killed *L. casei* IMAU60214.

## 4. Discussion

Nutritional imbalance has an important impact on the effector immune function and plays a role in the physiology and homeostasis of the host [40]. Several studies have shown that changes in nutritional status impact immune cellular metabolism, and these changes are associated with immunosuppression, which leads to susceptibility to infection [41]. Malnutrition is one cause of immunosuppression, which can complicate the satisfactory resolution of an infection, as it modifies the innate and adaptive immune response [42,43]. Previous studies in experimental animal models both in vivo and in vitro have demonstrated a deficiency in the activation mechanisms of macrophages [44]; however, the molecular mechanisms by which the macrophage fails in its immune function are still not well known. Mononuclear phagocyte cells—as monocytes circulating peripheral blood—and macrophage cells are essential components of innate immunity that play an important role in the elimination of pathogens [45].

Studies in rats with severe malnutrition suggested that the increase in the susceptibility of infection is largely attributable to the functional defects of mononuclear and polymorphonuclear cells, whereby, in hosts, immunocompetence conditions develop an important function of the activation of the early immune response and the connection of the adaptive immune response in its function as an antigen-presenting cell (such as om the case of MDMs) [19,46]. However, very few studies have supported the role and deregulation of these cells in children with malnutrition.

In this work, we analyze the effect of heat-killed *L. casei* IMAU60214 on the function of MDM cells obtained from malnourished infected (MNI) children, and this was compared with well-nourished infected (WNI) children. In addition, we studied a group of well-nourished healthy (WNH) children as a control group. Differences observed in the effector function of the untreated MDM cells of the three groups might reflect two facts: first, the comparison of the results obtained from WNH and WNI children reveals various changes that could be associated with the infection process, since the WNI children showed slightly elevated levels in the basal production of ROS, interleukin release, more phagocytic activity and bactericidal activity compared with WNH children; second, the differences observed between WNI and MNI children show changes related to nutritional condition.

On the other hand, when the MDM cells were pretreated with heat-killed *L. casei* IMAU60214, we observed an improved oxidative response with respect to untreated MDM cells in all the study groups. The increase of ROS production in MDM cells is essential because it is an important oxygen-dependent microbicidal mechanism; according to the data obtained, the pre-treatment with *L. casei* IMAU60214 could favor the control of infection [47]. In contrast to these results, previous studies have documented an absence of ROS secretion after stimulation with a probiotic strain; with respect to this, it has been documented that lactic acid bacteria strains secrete anti-oxidant components such as the superoxide dismutase enzyme which act on ROS, generating species with less oxidative activity [48]. This enzyme has been detected in viable LAB supernatants in culture [49]. The differences found in our study may be attributable to the non-viable condition (heat-killed) of *L. casei* IMAU60214.

Other relevant data found in our study include the increase in levels of TNF-α produced by stimulation with zymosan on MDM cells pre-treated with *L. casei* IMAU60214 with respect to untreated MDM cells. TNF-α is an important macrophage-activating cytokine that acts together with other cytokines including IFN-Υ; combined with the monocyte-macrophage colony-stimulating factor (M-CSF), these are the main cytokines that favor ROS secretion [17,50]. Likewise, we found that one of the pro-inflammatory cytokines that was released in higher levels was IL-6 on MDM cells pre-treated with *L. casei* IMAU60214 with respect to untreated MDM cells. This result was observed in the three groups. IL-6 is a cytokine with a regulatory function that favors the secretion of important acute phase proteins mediating the inflammatory response; IL-6 also modulates the adaptive immune response through the activation of B lymphocytes [51]. Another pro-inflammatory cytokine that was also increased after pretreatment with heat-killed *L. casei* IMAU60214 was IL-1β. This cytokine, IL-1β, is a pyrogen inducer that stimulates fever and leukocyte migration, as well as the polarization of subpopulations of T lymphocytes promoting T-helper 17 (Th17) cells [52]. In addition, this increase is related to the elimination of microorganisms through the activation of phagocytic cells.

In addition, we analyzed the effect of heat-killed *L. casei* IMAU60214 on MDM cells on the secretion level of IL-10—a cytokine with anti-inflammatory properties. The results showed increased levels of IL-10 produced by zymosan-stimulated MDM cells pre-treated with *L. casei* IMAU60214 with respect to untreated MDM cells in all groups. However, IL-10 showed a lower concentration with respect to pro-inflammatory cytokines (TNF-α, IL-1β and IL-6). IL-10 has an important homeostasis function, which is usually activated late in comparation with TNF-α, IL-1β and IL-8 [53]. These differences found in the levels of cytokine stimulation could suggest that the role of heat-killed *L. casei* IMAU60214 is related to an immunostimulatory activity instead of an immunomodulatory activity for MDM cells.

With respect to the phagocytic function of MDM cells, is well known that, during the process of the in vitro activation of macrophages, phagocytosis is an important immunological indication of the function of these cells [54]. For this reason, we analyze the phagocytic activity of the MDM cells in response to stimulation with zymosan before and after pre-treatment with heat-killed *L. casei* IMAU60214. An increase in phagocytic activity was observed in all groups of MDM cells pre-treated with heat-killed *L. casei* IMAU60214 with respect to untreated MDM cells. These results suggest that immunological ability is increased in MDM cells pre-treated with heat-killed *L. casei* IMAU60214.

Finally, we analyze the intracellular killing capacity of MDM cells from WNH, WNI and MNI children without pre-treatment and with pre-treatment with heat-killed *L. casei* IMAU60214. After confirming that MDM cells pre-treated with heat-killed *Lactobacillus* are able to increase phagocytic activity, we would like to determine whether MDM cells could kill pathogenic bacteria such as *S. typhimurium*, *E. coli* and *S. aureus*. The CFU/mL recovery of bacteria was reduced when the MDM cells were pre-treated with heat-killed *L. casei* IMAU60214 with respect to untreated MDM cells; this was observed in all study groups.

These results found in our work are in accordance with experimental murine models with malnutrition and respiratory infection by *Streptococcus pneumoniae*, where a decrease in the CFU/mL of *S. pneomoniae* after treatment with non-viable *L. rhamnosus* CRL1505 was shown, suggesting an improvement and recovery of mice with malnutrition and infection [55,56]. Likewise, previous reports have suggested that in malnourished mice nasally treated with both viable and heat-killed *Lactobacillus casei*, there was an enhanced immune response to pneumococcal infection, since they recovered less CFU/mL of pathogen after treatment [57], and anenhanced phagocytic activity of macrophages was found in male Sprague–Dawley rats with uremia, suggesting that *L. casei* restores the function of these cells [58]. Furthermore, it has been reported that oral administration of *L. acidophilus* and *Bifidobacterium* increases the ROS production and phagocytic activity of macrophages in old mice [59]. In a recent in vivo study, it was shown that feeding with probiotic *Lactobacillus reuteri* LR6 increases the phagocytic activity of the macrophages and bone marrow-derived dendritic cells (DCs); this strain acts as an adjuvant in the improvement of immune parameters using protein energy malnourished (PEM) murine models [60]. Another in vivo study performed with heat-killed probiotic showed a capacity to induce secretory IgA production in pre-term infants treated with a formulation containing heat-killed *Bifidobacterium breve* C50 and *Streptococcus thermophilus* [61]. The administration of heat-killed probiotics avoids the risks of oral use of live probiotics, including in cases of systemic infections due to translocation, particularly in vulnerable patients, malnourished infected children and oncology patients. This has increased interest in the study and proposal of non-viable microorganisms or microbial cell extracts for use as probiotics—mainly, heat-killed probiotic bacteria [23,62].

All the results obtained in our study suggest that the pre-treatment of MDM cells with heat-killed *L. casei* IMAU60214 makes an improvement of the recovery of the effector function of macrophages in children with malnutrition. Based on the above, we consider that the administration of heat-killed *Lactobacillus casei* IMAU60214 by adjuvant therapy, together with oral hydration solutions, could help malnourished patients with conditions complicated by diarrhea, who represent a high risk of mortality with respect to malnourished patients without diarrhea during their stay in Hospital. These conditions are in agreement with the previous reports that suggested that heat-killed bacteria retain immunostimulatory properties [63,64] and their function as potential bioactive foods in the prevention of infectious diseases in children [65]. However, further research is necessary to extend these observations with well-controlled clinical studies before their acceptance as a recommendation for alternative therapy. Our results should be interpreted with care, with an increase in sample size being necessary. In addition, the limitations of this study include the lack of adequate controls such as MDM cells only being stimulated with heat-killed *L. casei* IMAU60214 and the in vitro manipulation of monocytes not being differentiated to mature macrophages; thus, our work may not reflect the function of the host resident macrophages. Furthermore, the clinical population studied is heterogeneous and may not be representative of usual hospitalized patients, since the sample size studied was small for each of the groups, which does not allow us to make comparisons between clinical groups.

## 5. Conclusions

The present work demonstrated that heat-killed *L. casei* IMAU60214 is a potential candidate for regulating the immune function of macrophages; however, our study showed different degrees of effects on the groups studied, thus setting the biological basis of the beneficial effects of these non-viable probiotic bacteria in humans. These results are encouraging in terms of increasing the defenses of undernourished children. However, upcoming studies are necessary to allow the translation of this research to the possibility of alternative therapeutics in clinical research.

## Figures and Tables

**Table 1:** Demographic characteristics of children enrolled in this study.

Characteristics Cohort	Well-Nourished Healthy Children	Well-Nourished Infected Children	Malnourished Infected Children
Patients (*N)*	15	15	15
Girls/Boys, *n*	7(46%)/8(53%)	6(40%)/9(60%)	3(20%)/12(80%)
Age (months)	16.4 (±6.4)	15.7 (±7.5)	14.4 (±6.1)
Weight (kg)	12.5 (±0.5)	11 (±0.3)	5.4 (±0.2)
Height (cm)	83 (±1.2)/92.5 (±3)	82 (±3.4)/87.5 (±2.5)	75 (±2)/80 (±3.4)
Z-score (WHZ)	Z > −1 to Z < 1	Z > 0 to Z < 1	Z < −3
Clinical malnutrition ^a^	Normal	Normal	Severe malnutrition
(Non-edematous marasmus)	-	-	15
Respiratory tract infection	-	12	11
Multiple abscesses	-	1	-
Diarrhea	-	-	3
Periorbital cellulitis	-	1	-
Bacterial meningitis	-	1	1

^a^ Severity malnutrition was evaluated according to World Health Organization (WHO) parameters such as the weight-for-height Z-Score (WHZ) and height and weight data according to age. The data of weight and height are presented as the means ± standard deviation (*n* = 15).
